# Predictors and clinical impact of postoperative diarrhea after colorectal cancer surgery: a prospective, multicenter, observational study (SHISA-1602)

**DOI:** 10.1007/s00384-022-04097-8

**Published:** 2022-01-26

**Authors:** Hiroyuki Ohta, Toru Miyake, Tomoyuki Ueki, Masatsugu Kojima, Masayasu Kawasaki, Takeshi Tatsuta, Takekazu Iuchi, Sumihiro Kamitani, Tomoharu Shimizu, Eiji Mekata, Masaji Tani

**Affiliations:** 1 Department of Comprehensive Surgery, Shiga University of Medical Science, Seta-tsukinowacho, Otsu, Shiga 520-2192 Japan; 2 Department of Surgery, Higashi-Ohmi General Medical Center, Higashi-ohmi, Japan; 3 Department of Gastrointestinal Surgery, Shiga University of Medical Science, Otsu, Japan; 4 Department of Surgery, Bellland General Hospital, Sakai, Japan; 5 Department of Surgery, Tesseikai Neurosurgical Hospital, Sijounawate, Japan; 6 Department of Surgery, Toyosato Hospital, Toyosato, Inukami-gun Japan; 7 Department of Surgery, Kohnan Hospital, Koka, Japan; 8 Department of Medical Safety, Shiga University of Medical Science, Otsu, Japan

**Keywords:** Diarrhea, Complications, Colorectal cancer, Colorectal surgery

## Abstract

**Purpose:**

Postoperative diarrhea, including high-output stoma (HOS), frequently occurs after colorectal surgery; its risk factors and clinical implications on subsequent complications remain unknown. This study aimed to evaluate the risk factors and clinical implications of postoperative diarrhea after primary colorectal cancer (CRC) surgery.

**Methods:**

This prospective observational study included patients with CRC who underwent radical surgery at six hospitals between June 2016 and December 2017. The patients were categorized into three groups (non-stoma, colostoma, and ileostoma groups).

**Results:**

A total of 178 patients participated in the study. In the non-stoma group, the incidence of postoperative diarrhea was 18.4% (27/147). The incidence of HOS was 28.6% (4/14) in the ileostoma group, and 0% in the colostoma group. Multivariable analyses of the incidence of diarrhea in the non-stoma group indicated that habitual smoking and hypertension were significantly associated with postoperative diarrhea (*P* = 0.012 and *P* = 0.0274, respectively). Postoperative diarrhea was more likely to occur in patients with rectal cancer than in those with colon cancer (*P* = 0.0501). In the non-stoma and ileostoma groups, the probability of the occurrence of other complications with Clavien–Dindo (C-D) grades II or higher was significantly higher in patients with C-D grade I diarrhea, including HOS, than in patients without diarrhea (39.3% vs. 14.6%, *P* = 0.0061).

**Conclusions:**

Smoking and hypertension are the independent predictors of postoperative diarrhea after an elective CRC surgery. Rectal cancer surgery seems to be associated with postoperative diarrhea more than colon cancer surgery does. Mild postoperative diarrhea may lead to more severe complications.

## Introduction

Postoperative diarrhea is a frequent complication that can negatively affect the nutritional status and clinical outcome of patients after colorectal cancer (CRC) surgery. Various causes of postoperative diarrhea include bowel stenosis due to a huge tumor, surgical procedures, and perioperative pharmacological treatment [1, 2]. *Clostridioides difficile* (formerly known as *Clostridium difficile)* infection (CDI) is also a common nosocomial enteritis that causes diarrhea and occasionally severe symptoms [3, 4]. Meanwhile, excessive output from a stoma, called high-output stoma (HOS), can cause dehydration, renal dysfunction, and electrolyte abnormalities; it is also often observed in patients with stoma after CRC surgery [5, 6]. According to recent reports, HOS occurs in 16–43.3% of the patients who have undergone rectal resection and have a diverting ileostomy [5–7].

Over the last decade, many retrospective studies have been conducted to elucidate the chronic gastrointestinal complications after a CRC surgery, including diarrhea [2, 8, 9]. However, to date, only a few reports on prospective studies on acute postoperative diarrhea after CRC surgery have been published. The aim of this study was to elucidate the predictive factors of acute postoperative diarrhea and their relevance to complications after elective surgery in patients with primary CRC.

## Methods

### Study design

This (Shiga Surgical Association-1602) is a multicenter, prospective, observational study involving patients with postoperative diarrhea who underwent CRC surgery at six hospitals in Japan. A total of 178 consecutive patients with CRC, who were scheduled for an elective radical surgery between June 2016 and December 2017, were enrolled in this study. The study was performed in accordance with the ethical standards as laid down in the 1964 Declaration of Helsinki, and was approved by the Research Ethical Committee of Shiga University of Medical Science (approved IRB number: 27–222). Written informed consent was obtained from all eligible patients.

The inclusion criteria were as follows: (1) diagnosis of histopathologically confirmed CRC, (2) scheduled for curative resection, (3) age ≥ 20 years at the time of enrollment, and (4) performance status (PS) ≤ 2 on the Eastern Cooperative Oncology Group (ECOG) scale. Those with diarrheal symptoms before surgery, a history of CDI, or an emergency operation were excluded. Patients with severe complications or a PS ≥ 3 on the ECOG scale were also excluded, due to the likelihood of fecal incontinence. Bowel preparation before the surgery was performed at the discretion of each facility. This trial was registered with the UMIN Clinical Trials Registry (UMIN 000,022,206). This manuscript has been prepared in accordance with the STROBE guidelines [10].

### Evaluation of postoperative diarrhea and subsequent complications

The patients were categorized into three groups according to stoma creation: 1) non-stoma group, 2) colostoma group, and 3) ileostoma group. The definition of postoperative diarrhea for the non-stoma group in this study was adopted on the basis of the World Health Organization’s criteria; accordingly, postoperative diarrhea was defined as a “frequent watery bowel movement for more than three times per day within postoperative 30 days [11]”. Diarrhea corresponded to type 6 (mild; fluffy pieces with ragged edges, a mushy stool) and type 7 (severe; watery, no solid pieces, and entirely liquid) on the Bristol stool chart [12]. Meanwhile, in the colostoma and ileostoma groups, an HOS was defined as an ostomy excretion of greater than 2000 mL per day within postoperative 30 days. If postoperative diarrhea or HOS was diagnosed, a *Clostridioides difficile* (CD) toxin assay and fecal culture examinations were performed at the discretion of the attending physician. Other adverse events and postoperative complications, including surgical site infection (SSI), were also assessed according to the Clavien–Dindo (C-D) classification system [13].

We analyzed the patients’ background characteristics (sex, age, body mass index [BMI], PS, smoking habits, comorbidities [hypertension and diabetes mellitus], tumor location, and TNM classification stage [from the eighth edition of the Japanese Classification of Colorectal Carcinoma]) and preoperative care factors (therapeutic antibiotics, antacid, chemotherapy, hospital stay, mechanical preparation, and chemical preparation). Data on the postoperative outcomes (including the onset date and treatment of postoperative complications) and postoperative hospital days were prospectively collected. Analyses of risk factors for postoperative diarrhea were performed for the non-stoma group.

Furthermore, the clinical impact of mild postoperative diarrhea on other complications that followed was evaluated. The associations between C-D grade I diarrhea, including HOS, and other complications of C-D grades II and higher were investigated in the non-stoma and ileostoma groups; in both groups, the patients received bowel reconstruction. In this investigation, the patients with diarrhea of C-D grade II or higher were excluded. Furthermore, the patients who had diarrhea after other C-D grade II or higher complications were excluded in consideration of therapeutic antibiotic-induced diarrhea.

### Statistical analysis

The categorical variables are expressed as numbers and percentages. In the univariate analysis, group comparisons were performed using the Fisher’s exact test and the chi-squared test for binary variables. Multivariable logistic regression analysis was performed using variables found to be significant (*P* < 0.05) in the univariate analysis; factors predictive of postoperative diarrhea were determined. The analysis was performed using the backward stepwise regression method. A *P*-value of < 0.05 was considered to indicate statistical significance. Statistical analyses were carried out using the statistical program EZR (Saitama Medical Center, Jichi Medical University, Saitama, Japan), which is a graphical user interface for R (The R Foundation for Statistical Computing, Vienna, Austria) [14].

## Results

### Patients’ clinical background

A total of 178 patients were enrolled in this study; of these, 147 were categorized into the non-stoma group. Based on the stoma-creation surgery, 17 and 14 patients were categorized into the colostoma and ileostoma groups, respectively. Table 1 demonstrates the characteristics of the patients in the three groups. All the patients in the colostoma and ileostoma groups had permanent colostomy and diverting loop ileostomy, respectively. The entire study sample comprised 96 men and 82 women. The median age was 71 years, and the median BMI was 22 kg/m^2^; 72 patients (40.4%) were smokers, 69 patients (38.8%) had a high blood pressure, and 28 (15.7%) had diabetes mellitus. Preoperative medications were as follows: therapeutic antibiotics (*n* = 12, 6.7%), antacid (*n* = 32, 18.0%), and chemotherapy (*n* = 11, 6.2%). No patients underwent perioperative radiation therapy. The median preoperative hospital stay duration was 3 days. Before the surgery, 157 patients (88.2%) underwent mechanical bowel preparation, while 61 patients (34.3%) underwent chemical bowel preparation.

**Table 1:** Clinical characteristics of patients

Characteristics	Non-stoma group (*n* = 147)	Colostoma group (*n* = 17)	Ileostoma group (*n* = 14)
*Patient-related factors*			
Gender (Male:Female)	75:72	13:4	8:6
Age (years, median) [range]	72 [23–96]	73 [56–92]	67 [39–100]
BMI (kg/m^2^, median) [range]	22.1 [14.2–37.8]	20 [14.5–27.1]	22.5 [17.6–31.6]
ECOG PS 0/1/2	68/33/19	8/5/4	8/4/2
Smoking habits (*n*, %)	55 (37.4)	9 (52.9)	8 (57.1)
Hypertension (*n*, %)	60 (40.8)	3 (17.6)	6 (42.9)
Diabetes mellitus (*n*, %)	23 (15.6)	2 (11.8)	3 (21.4)
*Preoperative care-related factors*			
Preoperative therapeutic antibiotics (*n*, %)	10 (6.8)	1 (5.9)	1 (7.1)
Preoperative antiacid (*n*, %)	25 (17.0)	4 (23.5)	3 (21.4)
Preoperative hospital stay (days, median) [range]	3 [1–47]	6 [2–57]	4 [2-14]
Preoperative chemotherapy (*n*, %)	3 (2.0)	6 (35.3)	2 (14.3)
Mechanical preparation (*n*, %)	131 (89.1)	12 (70.6)	14 (100)
Chemical preparation (*n*, %)	49 (33.3)	7 (41.2)	5 (35.7)
*Tumor/surgery-related factors*			
Location	Right: 60/Left: 41/Rectum: 46	Left: 1/Rectum: 15/Right and rectum: 1	Rectum: 14
Procedure	ICR: 10/RHC: 47/TC: 2/LHC: 6/SC: 36 HAR: 31/LAR: 15	SC: 1/Hartmann: 5/APR: 8/TPE: 2 RHC + Hartmann: 1	LAR: 12/ISR: 2
TNM Stage 0/I/II/III/IV	1/38/27/62/19	0/0/4/7/6	0/7/1/5/1
Approach (Open:Laparoscopy)	39:108	5:12	7:7
Operation time (minutes, median) [range]	254 [26–487]	313 [187–889]	308.5 [175–550]
Blood loss (mL, median) [range]	40 [0–1510]	520 [20–3938]	170 [0–600]
Intraoperative transfusion (*n*, %)	4 (2.7)	7 (41.2)	0 (0)

*BMI* body mass index, *ICR* ileocecal resection, *RHC* right hemicolectomy, *TC* partial transverse colectomy, *LHC* left hemicolectomy, *SC* sigmoid colectomy, *HAR* high anterior resection, *LAR* low anterior resection, *ISR* intersphincteric resection, *APR* abdominoperineal resection, *TPE* total pelvic excenteration

The tumor locations were as follows: right colon (*n* = 60), left colon (*n* = 42), rectum (*n* = 75), and both the right colon and the rectum (*n* = 1). The TNM classification stages were as follows: 0 (*n* = 1), I (*n* = 45), II (*n* = 32), III (*n* = 74), and IV (*n* = 26). Among the 178 patients, 127 (71.3%) underwent laparoscopic surgery. The median operation time and blood loss were 240 min and 50 mL, respectively. Eleven patients (6.2%) received blood transfusion during the operation.

### Clinical features of postoperative diarrhea

In the non-stoma group, the incidence of postoperative diarrhea was 18.4% (27/147). Among the stoma groups, HOS occurred with an incidence of 28.6% (4/14) in the ileostoma group, while it did not occur in the colostoma group. Out of the 31 patients with postoperative diarrhea including HOS, 17 patients (54.8%) underwent the rapid CD toxin assay and fecal culture examination. All the patients tested negative for the CD toxin. However, two patients (11.8%) demonstrated CD-antigen positivity; one of these also had a positive fecal culture on examination. Both patients were clinically diagnosed with mild CDI (one on the third postoperative day and the other on the fourth postoperative day). They received metronidazole, in accordance with the clinical practice guidelines [15].

Twenty-nine patients with diarrhea due to causes other than CDI were all mildly ill and did not require special treatments, such as antibiotics; hence they were judged to have a C-D grade I postoperative complication. The median onset of diarrhea was postoperative day 4 (range, postoperative days 1–20). The median duration of postoperative hospitalization was 13 days in both patients who had diarrhea (range, 10–58 days) and those who did not (range, 7–90 days). In this study, patients with diarrhea did not take any postoperative aperients before the onset of diarrhea.

### Predictors of postoperative diarrhea in the non-stoma group

In the non-stoma group (*n* = 147), univariate analysis of various clinical factors for postoperative diarrhea was performed (Table 2). In the stoma group, the incidence and number of patients with HOS were too low for the analysis of HOS predictors. Smoking habits, hypertension, and tumor location (rectum) were significantly associated with postoperative diarrhea (*P* = 0.0146, *P* = 0.0497, and *P* = 0.042, respectively.)

**Table 2:** Univariate analysis of candidate risk factors for postoperative diarrhea in the non-stoma group (*n* = 147)

	Variables	Diarrhea (*n*)	Incidence (%)	*P* value
*Patient-related factors*				
Gender	Male	16/75	21.3	0.398
	Female	11/72	15.3	
Age	< 75	17/90	18.9	1
	≥ 75	10/57	17.5	
BMI	< 25	19/115	16.5	0.305
	≥ 25	8/32	25	
PS	0 · 1	23/124	18.5	1
	2	4/23	17.4	
Smoking habits	Yes	16/55	29.1	**0.0146**
	No	11/92	12	
Hypertension	Yes	16/60	26.7	**0.0497**
	No	11/87	12.6	
Diabetes mellitus	Yes	7/23	30.4	0.139
	No	20/124	16.1	
*Preoperative care-related factors*				
Preoperative therapeutic antibiotics	Yes	2/10	20	1
	No	25/137	18.2	
Preoperative antiacid	Yes	6/25	24	0.407
	No	21/122	17.2	
Preoperative hospital stay (days)	< 7	17/109	15.6	0.151
	≥ 7	10/38	26.3	
Preoperative chemotherapy	Yes	0/3	0	1
	No	27/144	18.8	
Mechanical preparation	Yes	24/131	18.3	1
	No	3/16	18.8	
Chemical preparation	Yes	6/49	12.2	0.258
	No	21/98	21.4	
*Tumor/surgery-related factors*				
Location	Colon	14/101	13.9	**0.042**
	Rectum	13/46	28.3	
TNM Stage	Early (0 · I)	6/39	15.4	0.638
	Advanced (II · III · IV)	21/108	19.4	
Approach	Open	7/39	17.9	1
	Lap	20/108	18.5	
Operation time (minutes)	< 300	23/125	18.4	1
	≥ 300	4/22	18.2	
Blood loss (mL)	< 200	22/121	18.2	1
	≥ 200	5/26	19.2	
Intraoperative transfusion	Yes	0/4	0	1
	No	27/143	18.9	

Numbers in bold represent statistically significant findings

The multivariable analysis included the three variables that showed statistical significance in the univariate analyses, i.e., smoking habits, hypertension, and tumor location (rectum). Among these, smoking habits (odds ratio [OR], 3.14; 95% confidence interval [CI], 1.29–7.66; *P* = 0.012) and hypertension (OR, 2.74; 95% CI, 1.12–6.70; *P* = 0.0274) were the independent predictive factors for postoperative diarrhea (Table 3). Rectal cancer surgery tended to be a statistically significant predictive factor (*P* = 0.0501).

**Table 3:** Multivariable analysis of risk factors for postoperative diarrhea in the non-stoma group

Variables	Odds ratio	95% CI	*P* value
Smoking habits (yes/no)	3.14	1.29–7.66	**0.012**
Hypertension (yes/no)	2.74	1.12–6.70	**0.0274**
Location (rectum/colon)	2.45	1.00–6.02	0.0501

CI, confidence interval

Numbers in bold represent statistically significant findings

### The relationship between postoperative diarrhea and other complications that followed

Finally, we investigated the impact of C-D grade I diarrhea on other complications of C-D grade II or higher. During the study period, no C-D grade IV life-threatening complications or C-D grade V complications (such as surgery-related deaths) were noted. There were no cases where C-D grade II and III complications occurred simultaneously. In this investigation, a patient with antibiotic-induced postoperative diarrhea after incisional SSI was excluded. Furthermore, two patients with C-D grade II CDI were excluded. The total incidence of C-D grade II and III complications in patients with C-D grade I diarrhea was 39.3% (11/28); this was significantly higher than that in patients without postoperative diarrhea (14.6% [19/130], *P* = 0.0061; Table 4). The details of the C-D grade II and III complications in patients with and without C-D grade I diarrhea are shown in Table 4. Among them, urinary tract infection (UTI) occurred a day after the onset of postoperative diarrhea in two men (7.1%; 2/28) with rectal cancer and a smoking habit. On the other hand, 130 patients without postoperative diarrhea were not significantly affected by UTI (*P* = 0.0305).

**Table 4:** Postoperative complications in the non-stoma and the ileostoma groups (*n* = 158)

Complications	With mild diarrhea (*n* = 28)	Without diarrhea (*n* = 130)	*P* value
Total	11 (39.3)	19 (14.6)	**0.0061**
C-D grade II	7 (25)	15 (11.5)	0.0741
C-D grade III	4 (14.3)	4 (3.1)	**0.0337**
Incisional surgical site infection	2 (7.1)	6 (4.6)	0.632
Ileus	2 (7.1)	4 (3.1)	0.288
Anastomotic leakage	1 (3.6)	3 (2.3)	0.546
Intra-abdominal abscess	1 (3.6)	1 (0.8)	0.324
Neurogenic bladder	1 (3.6)	0 (0.0)	0.177
Delirium	1 (3.6)	0 (0.0)	0.177
Heart failure	1 (3.6)	0 (0.0)	0.177
Urinary tract infection	2 (7.1)	0 (0.0)	**0.0305**
Pneumonia	0 (0.0)	2 (1.5)	1
Chyle leak	0 (0.0)	1 (0.8)	1
Port site hernia	0 (0.0)	1 (0.8)	1
Postoperative transfusion	0 (0.0)	1 (0.8)	1

Values are presented as numbers (%)

Numbers in bold represent statistically significant findings

*C-D* Clavien-Dindo

## Discussion

Postoperative diarrhea frequently occurs after CRC surgery and may have a harmful influence on the clinical course. However, postoperative diarrhea generally tends to be disregarded because of oblique clinical judgment and mostly mild symptoms. Our results suggest that smoking and hypertension are associated with postoperative diarrhea, although the definitive mechanism underlying postoperative diarrhea due to smoking and hypertension is unknown. Regarding cigarette smoking, some clinical analyses have shown that cigarette smoking is a risk factor for the development of inflammatory bowel disease (IBD), including Crohn’s disease, collagenous colitis, and lymphocytic colitis [16, 17]. Birrenbach and Böcker described potential mechanisms for the pathogenic interaction of nicotine and IBD, which include changes in the humoral and cellular immunity, cytokine levels, gut permeability, motility, and mucosal blood flow [16]. However, the relationship between intestinal motility and nicotine after colorectal surgery is still controversial [18]. On the other hand, a direct association between diarrhea and hypertension has never been described. Although enteropathy associated with consumption of some antihypertensive agents, such as beta-blockers and angiotensin II receptor blockers, has been recognized and reported so far [19, 20], in our study, the type of antihypertensive drug was not taken into consideration. However, clinicians should keep in mind that diarrhea could also be caused by some antihypertensive drugs.

Recently, some retrospective studies have reported that postoperative diarrhea impacts postoperative outcomes after colorectal surgery [21, 22]. Gaertner et al. described that postoperative diarrhea, whether in patients with CDI or not, was associated with superficial SSI [21]. Baker et al. reported that CDI significantly increased the likelihood of anastomotic leakage in patients with colectomy [22]. We previously reported that postoperative diarrhea occurring in the early phase might predict the development of symptomatic anastomotic leakage after laparoscopic low anterior resection [23]. From the results of this study, the patients with mild postoperative diarrhea experienced other severe complications (particularly postoperative UTI) more frequently as compared to patients without diarrhea. Regarding causal relationships, a possible interpretation of our findings is that dehydration and fecal contamination of the urinary tract coincided with postoperative diarrhea. However, only two patients developed UTI in our study, and the conclusive data were insufficient. Therefore, further large clinical trials may be expected. Meanwhile, postoperative diarrhea, including HOS, can lead to ileus and acute kidney injury (AKI) due to decreased intestinal motility and dehydration. In our investigation, there was no significant correlation of postoperative diarrhea with ileus or AKI of C-D grade II or higher.

Our data suggest that the patients who undergo rectal cancer surgery tend to experience postoperative diarrhea more commonly as compared to patients who undergo colon cancer surgery. However, this finding contradicts the findings of previous studies; right hemicolectomy is recognized to be associated with chronic postoperative diarrhea due to residual bile, which acts as an osmotic agent, and disruption of the ileal brake [2, 8, 24]. During right-sided hemicolectomy, we resected less than 10 cm of the terminal ileum as per the usual protocol. The physiological pathogenesis could differ between acute and chronic postoperative diarrhea after colorectal surgery. Decrease in the rectal reservoir and sacrifice of the hypogastric nerve plexus may influence not only a high frequency of defecation, but also acute diarrhea after rectal cancer surgery.

Perioperative oral synbiotic supplementation is expected to improve the digestive microbial balance and present postoperative infectious complications. Despite various reports on probiotics, prebiotics, and synbiotics, their efficacies in preventing CRC surgery complications remain controversial [25]. Komatsu et al. reported that the occurrence of postoperative complications did not differ significantly between the group taking synbiotics and the control group in a randomized controlled trial with 362 patients with CRC [26]. Meanwhile, Yang et al. reported that the incidence of diarrhea after CRC surgery was significantly lower in the probiotics group (26.7%, 8/30) than in the placebo group (53.3%, 16/30) [27]. According to our data, prophylactic oral administration of synbiotics for a week after surgery might be worth trying, especially in patients with rectal cancer with a smoking habit or hypertension.

There is no agreed definition of HOS; it refers to excretion ranging from 1500 to more than 2000 mL per day or for 2 consecutive days. The non-standardized definition in this study may have influenced the results. Moreover, this prospective study has several limitations, including the small number of patients, different operative procedures, absence of standardized bowel preparation, and difficulty in diagnosing CDI (due to the low sensitivity of the CD toxin assay). Finally, this study lacks a sufficient number of cases, which reduces the generalizability of its results. The courses and mechanisms of postoperative diarrhea are diverse and complex; therefore, further basic physiological and pathological studies may be needed along with clinical studies to identify ways to prevent postoperative diarrhea.

## Conclusion

Our findings suggest that smoking and hypertension are independent predictors of acute postoperative diarrhea, and mild postoperative diarrhea may lead to other more severe complications (including UTI) after an elective CRC surgery, especially rectal cancer surgery. Additional studies with much larger participant numbers are necessary to confirm the relationship between postoperative diarrhea and other complications in CRC surgery.
